# Editorial: Advancements in deep brain stimulation for chronic pain control

**DOI:** 10.3389/fpain.2023.1293919

**Published:** 2023-10-23

**Authors:** Michael D. Staudt, Nasser K. Yaghi, David J. Mazur-Hart, Prasad Shirvalkar

**Affiliations:** ^1^ Department of Neurosurgery, Beaumont Neuroscience Center, Royal Oak, MI, United States; ^2^ Department of Neurosurgery, Oakland University William Beaumont School of Medicine, Rochester, MI, United States; ^3^ Department of Neurosurgery, Barrow Neurological Institute, Phoenix, AZ, United States; ^4^ Department of Neurological Surgery, University of California San Francisco, San Francisco, CA, United States; ^5^ Department of Anesthesiology and Perioperative Care, Division of Pain Medicine, University of California San Francisco, San Francisco, CA, United States

**Keywords:** biomarker, chronic pain, deep brain stimulation, neuromodulation, neuropathic pain, transcranial magnetic stimulation

**Editorial on the Research Topic** Advancements in deep brain stimulation for chronic pain control

Chronic pain syndromes affect up to 20% of the population, impose significant suffering and are often resistant to available treatments (1, 2). Refractory chronic pain was among the first studied indications for brain stimulation over 50 years ago (3, 4). Deep brain stimulation (DBS) involves the reversible modulation of neural function by delivering electrical pulses to specific brain circuits through surgically implanted electrodes, which are connected to an implanted pacemaker device. Despite high rates of success reported in case reports and series using DBS for chronic pain, two large, sub-optimally designed clinical trials failed to meet primary endpoints in the 1990's (5). As a result, DBS for pain has been relegated to “off-label” or experimental use around the world, with no clear consensus on optimal brain targets, pain indications, or patient selection. To date, there has only been one rigorous, double-blinded randomized control trial testing DBS efficacy for chronic pain, which also failed to meet its primary endpoint (6). In parallel with a growing understanding of brain mechanisms for chronic pain in the last decade (7), DBS has undergone significant technological and scientific advancements for the treatment of approved neurological disorders such as epilepsy or Parkinson's disease. This Research Topic aims to integrate emerging insights into brain mechanisms of pain towards advancing DBS as viable therapy for chronic pain syndromes.

**Figure 1:**
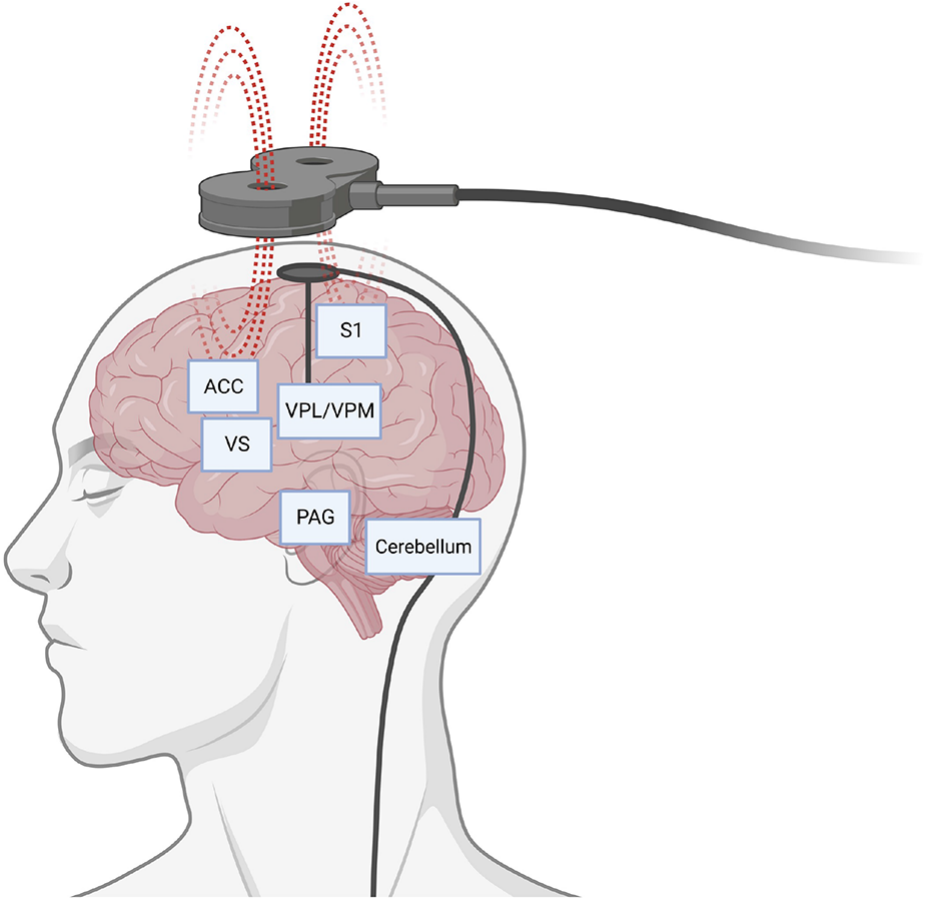
Representative display of potential brain circuits and targets for pain control through neuromodulation. Schematic depicts direct brain stimulation with an intracranial electrode or non-invasively with a transcranial magnet. ACC, anterior cingulate cortex; PAG, periaqueductal gray; S1, somatosensory area 1; VPL/VPM, ventral posterolateral and ventral posteromedial areas of the thalamus; VS, ventral striatum. Created with BioRender.com.

Pagano et al. reviewed the history of target selection for DBS, with a focus on the translational connection between preclinical and clinical studies. The periaqueductal/periventricular gray matter and sensory thalamic nuclei are considered classical targets, typically for the treatment of nociceptive and neuropathic pain syndromes, respectively. The authors review potential explanations for high variability of results between different preclinical and clinical studies, including the complexity of pain and the potential involvement of multiple networks (Figure 1).

There has been a more recent focus on targeting the affective and emotional components of pain with stimulation of the anterior cingulate cortex or the ventral striatum/anterior limb of the internal capsule, although similar challenges have been encountered in improving pain disability outcomes (6, 8). In the preclinical study by Liu et al., stimulation of the prelimbic area of the prefrontal cortex or the anterior cingulate cortex in rats was able to inhibit sensory and affective components of acute pain, although questions remain due to the non-specific stimulation of brain regions involved in extensive functions and connections.

A specific challenge in determining the “best” candidate patient or syndrome for DBS is that therapeutic responses to stimulation can be highly variable. No single target or set of stimulation parameters has proven efficacious for a specific pain syndrome across individuals. Motzkin et al. reviewed related challenges in understanding the neural basis for the uniquely individual aspects of pain, and propose a personalized approach via network-informed target selection. Identifying reliable biomarkers of pain is hoped to guide future therapies, as highlighted by Silva-Passadouro et al. — these authors evaluated frontal alpha-band asymmetry, which reflects the lateralization of prefrontal cortex activity, as a potential neurophysiological correlate of pain-related catastrophizing.

Non-invasive brain stimulation methods potentially provide a more accessible option to treat pain. Transcranial direct current stimulation and repetitive transcranial magnetic stimulation are two common non-invasive techniques studied for the treatment of neuropsychiatric disorders and chronic pain. The proposed mechanism of these modalities is via the modulation of cortical excitability and to facilitate neuroplasticity (9, 10). Alcon and Wang-Price reviewed the cognitive-affective deficits in chronic low back pain, and propose a potential synergistic role for cognitive therapies and non-invasive brain stimulation for the treatment of this prevalent pathology. Additional debilitating pain conditions include chronic migraines and headache, which have received increased attention using non-invasive brain stimulation (11, 12). As explored in the review by Noseda, there is increasing evidence to suggest a link between cerebellar networks and migraine pathophysiology. The cerebellum is known to influence motor and non-motor behaviors, and both invasive and non-invasive cerebellar stimulation has been proposed for the treatment of a multitude of neurological disorders (13).

This Research Topic showcases some of the most recent advancements in treatment for chronic pain centered on electrical stimulation of the brain and neuromodulation. There are areas in which gaps in knowledge remain, including physical perception of pain, social stressors, and affective components which can directly impact chronic pain control. Brain-computer interfaces will be important not only to study the origins of chronic pain within the brain, but also provide real-time modulation for treatment. Identifying new biomarkers for chronic pain will help further subtype this heterogeneous disease process and allow for more personalized treatment. Understanding network-level targets rather than individual targets in isolation will be important for treatments. Additionally, studying the long-term outcomes in pain reduction with current brain stimulation treatment modalities will be important to better understand habituation or loss of therapeutic efficacy to develop strategies to overcome this limitation. Likewise, advancements in engineering and hardware technology will further open this frontier as more precise and different types of electrical stimulation can be provided to new parts of the brain, via both invasive and non-invasive methods. Additionally, the advent of closed-loop systems will be able to identify neural inputs to direct adjustable outputs as seen in modulating motor symptoms in Parkinson's disease (14). The advent of artificial intelligence may allow rapid understanding of neural network signaling as seen in Parkinson's disease (15).

This Research Topic features both basic science and translational approaches to understanding and treating chronic pain. Chronic pain treatment will likely require a multi-modal approach to produce augmentative effects. The field will require rigorous investigations into the fields of neuroscience and electrical engineering. The first step will be requesting the aid of public officials to help prioritize this large, public need. Historical surgical approaches to psychotherapy lacked ethical and scientific rigor, and have left the field drastically behind other fields of medicine including other fields of neuroscience (16). Broader approval for closely regulated research would help future studies. Additionally, a validated biomarker for pain would help advance the field. This will require the cooperation of hospitals, universities, and industry to prioritize high quality research that emphasizes long-term patient outcomes. Study in this field of brain stimulation for chronic pain control remains important and highly impactful.
